# Thinner than a knife’s edge: 3D-printed liquid sheet jet technology for solution phase XFEL experiments

**DOI:** 10.1107/S2052252523009429

**Published:** 2023-11-01

**Authors:** Martin Trebbin

**Affiliations:** a University at Buffalo, Department of Chemistry & Research and Education in eNergy, Environment and Water (RENEW), 760 Natural Sciences Complex, Buffalo, NY 14260, USA

**Keywords:** free-electron lasers, XFELs, sample delivery, liquid sheets, solution phase, X-ray scattering

## Abstract

In this commentary, we explore the pioneering implementation of 3D-printed thin liquid sheet devices for advanced X-ray scattering and spectroscopy experiments at high-repetition rate XFELs.

The unique characteristics of X-ray free-electron lasers (XFELs) have opened new experimental frontiers for the life and materials sciences. This has enabled the development of novel methods, such as serial femtosecond crystallography (SFX), fluctuation X-ray scattering (FXS), and several X-ray spectroscopies, which have transformed atomic resolution molecular imaging and ultrafast materials characterization (Chapman *et al.*, 2011 ▸; Kraus *et al.*, 2018 ▸; Bergmann *et al.*, 2021 ▸; (Konold *et al.*, 2023 ▸). The XFELs’ ultrashort X-ray pulses with exceptional peak brilliance and spatial coherence allow us to record molecular movies of chemical and biological processes with unprecedented spatial and temporal resolution. These exciting possibilities come at a hefty price, however: the sample typically gets *blown up* or heavily modified by the intense X-ray dose (Neutze *et al.*, 2000 ▸; Stan *et al.*, 2016 ▸). Although the diffraction-before-destruction principle still allows damage-free data collection at room temperature at XFELs (Chapman *et al.*, 2011 ▸), the sample must be swiftly and efficiently replaced before the next X-ray pulse arrives. While the limited availability of most biological samples already complicates the technical realization of this requirement, the introduction of MHz-rate XFELs further exacerbates the challenge (Wiedorn *et al.*, 2018*b* ▸; Grünbein *et al.*, 2018 ▸; Sobolev *et al.*, 2020 ▸). Furthermore, the produced data must be of highest possible quality and collected with maximum efficiency regarding sample and beamtime use (Konold *et al.*, 2023 ▸). Thus, the effective and efficient investigation of liquid phase sample specimens with high-precision X-ray techniques still represents a unique challenge for XFELs (Konold *et al.*, 2023 ▸).

At present, the gas-dynamic virtual nozzle (GDVN) is predominantly utilized for liquid sample injection in XFELs (DePonte *et al.*, 2008 ▸; Vakili *et al.*, 2020 ▸; Vakili *et al.*, 2022 ▸, Knoška *et al.*, 2020 ▸; Nazari *et al.*, 2020 ▸; Nelson *et al.*, 2016 ▸; Trebbin *et al.*, 2014 ▸). In brief, a microscopic liquid stream is sheathed and accelerated by a stream of pressurized gas, forming a few-micron-wide liquid jet that is ejected in the vacuum (Gañán-Calvo, 1998 ▸; DePonte *et al.*, 2008 ▸). The resulting cylindrical liquid jet must reach velocities on the order of tens of m s^−1^ to outpace radiation-induced sample explosions from consecutively incoming X-ray pulses (Stan *et al.*, 2016 ▸; Wiedorn *et al.*, 2018*a* ▸; Wiedorn *et al.*, 2018*b* ▸). Today, these GDVNs are typically produced via high-resolution 3D printing, such as two-photon polymerization (Oberthuer *et al.*, 2017 ▸; Knoška *et al.*, 2020 ▸), yielding reproducible channel geometries that allow the minimization of sample flow rates, scattering background from the solvent, and reduce extreme sample consumption. However, this GDVN approach comes with longstanding issues severely impacting the success rate of XFEL experiments, such as vacuum compatibility issues, susceptibility to clogging, and large background fluctuations (Konold *et al.*, 2023 ▸). The latter challenge primarily stems from the X-ray beam pointing fluctuations hitting the cylindrical liquid jet: with typical jet diameters of *ca* 3 µm, slight deviations from the jet center result in significant changes of the X-ray interaction volume (Eggers & Villermaux, 2008 ▸).

An emerging method to overcome many of the above challenges is the use of liquid sheets that provide a consistent thickness over large areas (Ha *et al.*, 2018 ▸; Koralek *et al.*, 2018 ▸; Hoffman *et al.*, 2022*a* ▸; Hoffman *et al.*, 2022*b* ▸). Originally, free-flowing liquid sheets were generated using two impinging laminar jets resulting in smooth flows and stable jetting over long periods (Taylor, 1960 ▸; Bush & Hasha, 1999 ▸). More recently, glass-based microfluidic devices made via lithography were demonstrated utilizing gas acceleration to generate dramatically thinner sheets (Koralek *et al.*, 2018 ▸; Hoffman *et al.*, 2022*a* ▸; Hoffman *et al.*, 2022*b* ▸). However, the use of sheet jets at XFELs has been far less prevalent due to high liquid and gas loads that complicate vacuum operation (Konold *et al.*, 2023 ▸).

In the current issue of **IUCrJ** , Konold *et al.* present a refreshing and exciting approach addressing these above challenges: a 3D-printed gas-accelerating nozzle design for the generation of thin liquid sheets providing reproducible device fabrication, excellent stability, reduced liquid flow rates, low background and compatibility with high-repetition rate XFELs (Konold *et al.*, 2023 ▸). The device performance has been thoroughly investigated at the European XFEL’s SPB/SFX instrument using nanofocused X-ray scattering and highspeed video microscopy, yielding promising results for the interrogation of liquid samples at XFELs. Two of the most interesting results of this study are the large and even hit area and the achieved sheet thicknesses, as shown in Fig. 1 ▸. However, we will also shed light on some operational nuances that still have to be overcome.

First, the flatness and large area of the generated sheet vastly reduces sensitivity to X-ray beam pointing fluctuations improving scaling characteristics. The liquid sheet also overcomes longstanding difficulties of cylindrical jets, such as the distortion of pump laser focus properties or the calculation of take-off angles for X-ray photoelectron spectroscopy (Konold *et al.*, 2023 ▸).

Second, the liquid sheet jets can be made very thin, ranging from a few hundred nanometres down to 60 nm. Compared with traditional GDVN jets, the *ca* 30-fold reduction in X-ray path length and 4-fold reduction in background offers intriguing experimental possibilities (Konold *et al.*, 2023 ▸). Specifically, the background levels for X-ray scattering and spectroscopic investigations can be dramatically reduced, approaching the single-molecule scale. The minimal, wedge-like thickness variation can be used as a feature for swiftly adjusting the sample thickness on the fly by simply translating the nozzle. One must also not forget that the reduction in interaction volumes also reduces the impact from radiation-induced explosions, resulting in perturbation-free solution scattering data collection up to 564 kHz.

While the liquid flow rate is lower than previous sheet jet devices, it remains a challenge for scarce samples, such as proteins, because the liquid flow rate is still 3- to 5-fold higher than the GDVN it was compared with (Konold *et al.*, 2023 ▸). Consequently, the vacuum compatibility of these devices remains a challenge, typically requiring differential pumping, cryo-trapping, and/or heated sample catchers, which are not present at every beamline, thus, limiting applicability (Konold *et al.*, 2023 ▸). However, since most of the liquid volume is found in the much thicker rims, the authors make a few suggestions on how to potentially overcome these challenges, such as reducing channel dimensions or using carrier liquids, but those solutions will require further development (Hoffman *et al.*, 2022*a* ▸; Oberthuer *et al.*, 2017 ▸). Another aspect to consider is the parabolic velocity profile across the sheet jet resulting from the gas focusing, which might make time-resolved experiments triggered by rapid mixing challenging to evaluate (Choo & Kang, 2002 ▸).

Although the study and evaluation were carried out thoroughly and in detail, showcasing the exciting applications for sheet jets, it is noteworthy that the experiments have been carried out only with 2-propanol. Thus, it remains to be seen how these devices will perform under realistic conditions, *i.e.* with aqueous samples and buffers, which are more prone to icing and clogging, especially under vacuum conditions.

While there is still some room for improvement, the liquid sheet technology remains an exciting addition to the liquid phase sample delivery toolset at XFELs because the tremendous experimental opportunities far outweigh the remaining few challenges. The stable, low background and large flat area of liquid sheets down to tens of nanometres of thickness offer not only great opportunities for various (soft) X-ray spectroscopies (Ekimova *et al.*, 2015 ▸; Smith *et al.*, 2020 ▸; Fondell *et al.*, 2017 ▸), high-harmonic generation (Luu *et al.*, 2018 ▸) and high-intensity laser plasma investigations (George *et al.*, 2019 ▸), but also lead the way towards electron diffraction (Nunes *et al.*, 2020 ▸; Yang *et al.*, 2021 ▸) and small-/wide-angle X-ray solution scattering experiments (Blanchet *et al.*, 2023 ▸). The nanometre-thin liquid sheets with low, stable background are especially attractive for experiments which benefit from small interaction volumes, such as fluctuation X-ray scattering (Kurta *et al.*, 2017 ▸; Pande *et al.*, 2018 ▸; Konold *et al.*, 2023 ▸). With thicknesses approaching those of vitreous ice in cryo-electron microscopy, this study points towards the thrilling possibility of carrying out ultrafast single-particle solution scattering (Adrian *et al.*, 1984 ▸; Kontziampasis *et al.*, 2019 ▸; Klebl *et al.*, 2020*a* ▸; Klebl *et al.*, 2020*b* ▸).

## Figures and Tables

**Figure 1:**
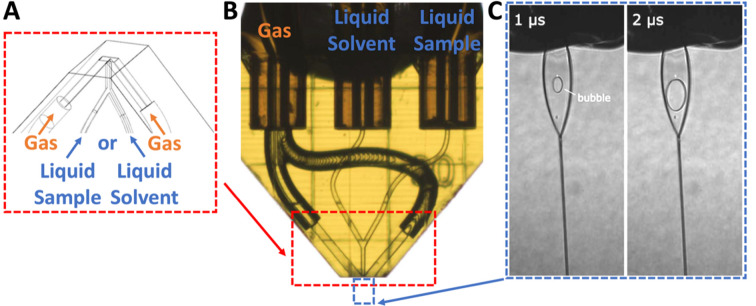
Konold *et al.* present a 3D-printed nozzle for the generation of sheet jets. (*A*) CAD-design of the gas-accelerated nozzle design. (*B*) A microscopic image of the 3D-printed nozzle (*ca* 1.5 mm long). (*C*) Stroboscopic images of sheet jet explosion and void formation from two consecutive XFEL pulses (141 kHz). Image adapted from Konold *et al.* (2023 ▸).
